# Inflammatory Response to Different Toxins in Experimental Sepsis Models

**DOI:** 10.3390/ijms20184341

**Published:** 2019-09-05

**Authors:** Kayle Dickson, Christian Lehmann

**Affiliations:** 1 Department of Microbiology and Immunology, Dalhousie University, Halifax, NS B3H 4R2, Canada; 2 Department of Physiology and Biophysics, Dalhousie University, Halifax, NS B3H 4R2, Canada; 3 Department of Pharmacology, Dalhousie University, Halifax, NS B3H 4R2, Canada; 4 Department of Anesthesia, Pain Management and Perioperative Medicine, Dalhousie University, Halifax, NS B3H 4R2, Canada

**Keywords:** inflammation, sepsis, endotoxemia, infection

## Abstract

Sepsis is defined as life-threatening organ dysfunction caused by the dysregulated host response to infection. Despite serious mortality and morbidity, no sepsis-specific drugs exist. Endotoxemia is often used to model the hyperinflammation associated with early sepsis. This model classically uses lipopolysaccharide (LPS) from Gram-negative pathogens to activate the immune system, leading to hyperinflammation, microcirculatory disturbances and death. Other toxins may also be used to activate the immune system including Gram-positive peptidoglycan (PG) and lipoteichoic acid (LTA). In addition to these standard toxins, other bacterial components can induce inflammation. These molecules activate different signaling pathways and produce different physiological responses which can be taken advantage of for sepsis modeling. Endotoxemia modeling can provide information on pathways to inflammation in sepsis and contribute to preclinical drug development.

## 1. Introduction

Sepsis is a complex clinical syndrome associated with significant morbidity and mortality. Sepsis has most recently been defined as a life-threatening organ dysfunction caused by the dysregulated host response to infection [1]. Sepsis has been hypothesized to affect 31 million people per year globally, resulting in over 5 million deaths, but these results are likely an underestimation of the true global impact of sepsis due to the unavailability of data from many low-income countries [2]. An increased risk of mortality persists after the initial episode of sepsis for over 2 years after hospital admission, which is potentially associated with secondary infections or vascular changes [3]. This increased mortality risk persists even when adjusting for the co-morbidities often seen in septic patients, such as diabetes mellitus [4]. Although outcomes are improving, there exists no approved treatment specifically for sepsis. This lack of treatment has led to a push in sepsis research, mainly focused on attenuating the hyperinflammatory state and microcirculatory disturbances associated with sepsis. 

Sepsis severity is determined in part by the pathogen responsible for the initial infection. Originally thought to result mainly from Gram-negative pathogens, current epidemiology now suggests a bigger role for Gram-positive pathogens [5]. Prompt antibiotic administration is essential for patient outcomes, and rapid pathogen identification can allow for the selection of appropriate spectrum antibiotics [6]. This proves challenging for clinicians as identifying the pathogen is fraught with difficulties including slow diagnostic testing, falsely negative results due to the administration of broad-spectrum antibiotics, and the evolution of resistant pathogens due to over-treatment with inappropriate antibiotics [7]. Next generation sequencing techniques make it possible to identify a pathogen in the blood stream in just thirty hours, but this is still far from the ideal, i.e. antibiotic administration within an hour of suspected sepsis [7]. As such, there is still a great need for effective therapies that are not dependent on knowledge of the pathogen. 

Septic patients have a confirmed or suspected infection by definition, which might be accompanied with endotoxemia if endotoxin is detectable in the blood stream. Endotoxins are released by micro-organisms during growth and destruction. They are potent inducers of a pro-inflammatory immune response. Lipopolysaccharide (LPS) of the Gram-negative bacterial membrane is the classical endotoxin. It is also the most well understood due to its established role in inflammatory pathways. Other bacterial components such as peptidoglycan (PG) and lipoteichoic acids (LTA) from Gram-positive bacteria can also cause systemic inflammation [8]. One meta-analysis demonstrated poor correlations between endotoxemia and Gram-negative bacteremia, which varied by pathogen [9]. This has reduced the utility of endotoxemia detection as a tool for the diagnosis of sepsis. 

**Figure 1:**
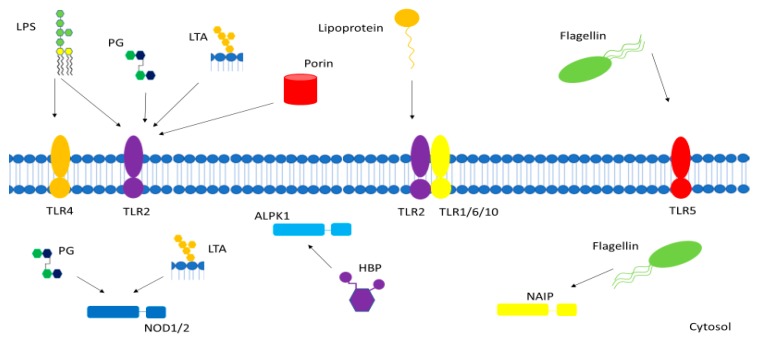
Gram-positive and Gram-negative bacterial pathogen-associated molecular patterns (PAMPs) interact with various membrane-bound and cytosolic receptors within the host cell. LPS—lipopolysaccharide, PG—peptidoglycan, LTA—lipoteichoic acid, TLR—toll-like receptor, NOD—nucleotide-binding oligomerization domain-containing protein, NAIP—NOD-like receptor apoptosis inhibitory protein, HBP—heptose-1,7-bisphosphate, ALPK1—alpha-kinase 1.

Endotoxemia can be used to model the acute inflammatory response associated with sepsis. Endotoxin administration is typically performed by intravenous/intraperitoneal injection or instillation of LPS to establish systemic inflammation. These models do not use living bacteria, so no active infection is established but host immune pathways are activated to provide insight on basic pathways. The host immune system uses pattern recognition receptors (PRRs) on immune sentinel cells to sense invading pathogens. Conserved bacterial structures are identified as foreign by several major receptor families, as discussed later in relation to specific pathogen-associated molecular patterns (PAMPs). LPS is the major Gram-negative PAMP. As such, much endotoxemia research has focused on using LPS as the toxin. Toxin administration induces systemic inflammation, leading to immune cell activation, release of inflammatory mediators and vascular/hemodynamic changes. The immune system is activated, and reactive oxygen species are produced, which can damage endothelial cells and lead to microcirculatory dysfunction and ultimately multi-organ failure. This resembles the immune activation seen in sepsis, but the magnitude of the response varies. Humans are considered to be very sensitive to endotoxin administration, but few studies compare human response to animal response [10]. PG and LTA can also activate the immune system via different pathways. Because of the differences in innate immune signaling pathways, it is reasonable to assume some differences will exist in the response when using different molecules for endotoxemia model. These differences may also surface at the microcirculatory level. An overview of these PAMPs and their various receptor is given in Figure 1. 

The mouse has historically been the animal of choice for experimental sepsis research, partially for its physiological similarities to a human. There is a high degree of genetic similarity between humans and mice despite differences in genome size [11]. Copeland et al. note the striking similarities between cytokine profiles in humans and mice [12]. Mice require a 250× higher endotoxin dose to reach human levels of IL-6 production, but most physiological responses are comparable [12]. Additional dosage considerations may be necessary when using alternative molecules to LPS, which is related to the different activation pathways [13]. Advantages of mouse models include low cost, reproducibility, and availability of knock out strains. While endotoxemia models lack the complexity seen in infectious models, they have ongoing utility in preclinical research specifically in examining inflammatory processes which occur in the initial stages of sepsis and mechanistic work. Knowledge of the mechanisms and signaling pathways involved in the pathogenesis of specific pathogens is critical for the development of novel therapies. Several of these pathways will be discussed in brief in this review, including activation by Gram-negative, Gram-positive, and other bacterial components. 

## 2. Gram-Negative Toxins

### 2.1. Lipopolysaccharide

LPS is the most common toxin used in endotoxemia modeling. Chemically, LPS consists of three distinct parts; the lipid A endotoxin, core oligosaccharide and O-antigen. The lipid A endotoxin is responsible for the toxicity of LPS in sepsis. The O-antigen region represents most of the variation between species of bacteria and gives the species-specific serotype. Other regions of LPS are relatively conserved. Lipid A is anchored in the outer membrane of Gram-negative bacteria. KDO (C-8 keto-deoxy-octonic acid) links the O-antigen to the core oligosaccharide. Sugar moieties make up the structural backbone of LPS, but the degree of acylation and phosphorylation may vary both within and across species. The four to five units of sugars in the O-antigen confer antigenic specificity between species. The lipid A and core oligosaccharide are essential for bacterial survival, but the O antigen may be missing. LPS with all requisite parts is termed smooth LPS (S-LPS). When the O-antigen is absent, it is referred to as rough LPS (R-LPS), named for the unique appearance of these bacteria when cultured. Deep rough mutants, where the core polysaccharide is also absent, are extremely susceptible to threats, including detergents, mutagens and antibiotics.

### 2.2. LPS Signaling

Immune signaling in response to LPS primarily involves the interaction of LPS with specific immune cell receptors. The Toll-like receptor (TLR) family is involved in triggering innate immune responses in mammals. Five TLRs have been described which contain extracellular leucine-rich repeats for recognition of bacterial PAMPS, and a cytoplasmic portion for intracellular signaling [14]. LPS activates Toll-Like Receptor 4 (TLR4) and myeloid-differentiation factor 2 (MD-2), an associated protein that is required for LPS signaling [14]. Serum lipopolysaccharide binding protein (LBP) and membrane-associated CD14 (mCD-14) act as accessory proteins to allow LPS to bind to its innate immune cell receptor. LPS binds to a hydrophobic pocket in MD-2 via an interaction with lipid A fatty acid chains [15]. LPS binding results in multimer formation, consisting of two copies of TLR4-MD-2 complex, and an LPS bridge [15]. Adaptor proteins are then recruited to the intracellular domain of the complex, which activates both MyD88-dependent and independent pathways. The TIRAP and MyD88 adaptor pair activate transcription factor NF-κB and mitogen-activated protein kinases (MAPKs), causing the production of pro-inflammatory cytokines. The receptor-LPS complex can also undergo clathrin and dynamin-mediated endocytosis and induce signaling from within the endosome via a second adaptor pair; TRAM and TRIF [16]. Most LPS-inducible genes are activated through this TRAM/TRIF pathway, including type 1 interferons, interferon-inducible genes and some chemokines. CD14 plays a role in both signal transduction pathways as it is involved in LPS trafficking and receptor internalization [16]. LPS signaling is clearly a highly involved process with many immune consequences which are of interest in sepsis. 

Antibiotic administration can facilitate the release of endotoxin by disrupting the bacterial membrane, complicating the treatment of sepsis. Antibiotic classes differ in their ability to cause lysis of the bacterial membrane and endotoxin release. Carbapenem antibiotics such as imipenem and meropenem have been associated with reduced endotoxin-release compared to ceftazidime [17]. Soluble LPS is known to be significantly more potent than membrane-bound LPS, so there may be benefit in reducing levels of released endotoxin [18]. Lepper et al. suggest this antibiotic property may have resulted in biased studies where there are variable endotoxin levels due to antibiotic-mediated release [19]. Endotoxemia models allow the examination of adjunct treatment options without the introduction of this additional variable. It remains important to consider possible variations in response when using LPS alone. One study notes a 10-fold decrease in neutrophil migration across the microvasculature in vitro when comparing purified LPS to intact *E. coli* [20].

### 2.3. Variations of LPS

Variations in LPS serotypes primarily affect potency, which causes a change in the immune response. LPS from *Helicobacter pylori* is significantly less toxic than LPS from Enterobacteriaceae with a 1000-fold reduction of pyrogenicity and mitogenicity and a 500-fold reduction in toxicity [21]. Lipid A from *H. pylori* is heavily modified with a reduced tetra-acyl lipid A structure, no ester-bound 4’-phosphate groups and an ethanolamine head group, leading to low endotoxin activity [22]. Further variation has been observed between serotypes of *H. pylori*, which can alter its LPS antigens to facilitate proliferation in a gastric environment prevent host recognition and destruction [23,24]. For example, removal of phosphate groups on the lipid A portion of LPS is essential for immune evasion with mutant bacteria unable to colonize the host [25]. The chemical structure plays a role in both pathogen survival and endotoxin activity for *H. pylori*.

Because of their impact on immune response, synthetic lipid A modifications have been assessed as a potential therapy for sepsis, specifically as TLR4 blockades to prevent inflammatory downstream effects [26]. Pre-clinical evidence on the efficacy of TLR4 modulation led to two large clinical trials. Eritoran is a synthetic Lipid A structure that antagonizes TLR4 signaling by blocking MD-2 binding and terminating the pathway [27]. These results were robust pre-clinically which led to a phase III clinical trial for sepsis [28,29]. Ultimately the clinical trial, known as the ACCESS randomized trial, failed for largely unknown reasons [30]. A weakly antagonistic LPS may have been a more appropriate choice, rather than complete antagonism, to control TLR4 activation [31]. TAK-242, a small molecule inhibitor of TLR4 that prevents interaction with adaptor proteins, also proceeded to clinical trials [32]. TAK-242 failed to suppress IL-6 production and did not significantly reduce mortality so the trial was terminated [33]. Despite pre-clinical evidence, TLR4 modulation has not been able to alter the inflammatory cascade in humans with sepsis. Based on these failures, most drug development has moved away from attempting to block this stage of the inflammatory action of LPS for therapeutic purposes. Research is ongoing to determine whether TLR4 modulation may still be useful in combination therapies, or in inflammatory conditions other than sepsis [32].

The majority of LPS serotypes signal through TLR4, but some may signal through an alternative pathway. *H. pylori* is an example of this, as LPS from this species primarily activates TLR2 with some strains able to antagonize TLR4 [22]. The level of antagonism possible is directly dependent on the ability of the pathogen to recruit TLR4 into lipid rafts based on differences in their lipid A structures [22]. TLR2 activation by *H. pylori* can also lead to differential expression of various claudins, which are tight junction proteins, based on TLR2 density and STAT3 activation [34]. Dysregulated claudin expression is involved in carcinogenesis associated with *H. pylori* infection [35]. LPS from the oral pathogen *Porphyromonas gingivalis*, which does not signal through TLR4, induces similar clonal expansion of CD4+ and CD8+ T cells when compared to LPS from *E. coli*, but different cytokine profiles result leading to variations in the adaptive immune response to these pathogens [36]. Different subsets of dendritic cells can influence the differentiation of T cells and ultimately the type of immune response occurring [37]. *P. gingivalis* has a distinct lipid A structure with unusually long and branched fatty acids, which results in less TLR4 signaling, activation of a different subset of DCs and a push towards a Th2 immune response when compared to LPS from *E. coli* [36]. *P. gingivalis*’ reduced signaling through TLR4 can likely be attributed to MD-2, which has the ability to discriminate between small variations in the chemical structure of lipid A. A mutation in TLR4 usually leads to endotoxin-tolerance, but these mutants are susceptible to Lipid A from *P. gingivalis*, which normally has a low endotoxin activity, as a different signaling pathway is activated [38,39].

Rough or smooth status of the LPS can also impact LPS signaling and the biological activity of these molecules. These differences may be due to differences in receptor interactions mediated by the changes in chemical components. CD36 is a scavenger receptor that can differentially activate macrophages in response to the presence of R-LPS or S-LPS and their concentration, as well as the presence of serum [40]. CD36 specifically acts to fine-tune macrophage response to the more virulent S-LPS and enable the host to sense lower levels of bacteria [40]. CD36 is also involved in negative regulation in later stages of infection, when serum is present, where it prevents an excessive pro-inflammatory response [40]. Some evidence suggests that R-LPS may be less dependent on CD14 signaling, but S-LPS is more efficient at inducing cytokine production [41]. 

LPS from *E. coli* is the most common choice for endotoxemia experiments, but other species and specific serotypes could be used. Depending on the nature of the experiment, detailed serotype knowledge may not be critical. The potency of each serotype of LPS should be evaluated individually due to possible differences in batches. Structural changes may impact outcome if virulence, immune evasion, or antibiotic resistance is impacted. As such, it may be beneficial to use a standardized LPS unless considering a unique element associated with a serotype. It must be acknowledged that a single serotype presents a narrow view of signaling pathway possibilities in sepsis. While many studies have utilized various types of LPS, few have attempted to draw direct comparisons between molecules from difference species or different serotypes.

## 3. Gram-Positive Toxins

Most sepsis research has focused on Gram-negative bacteria due to the well-established role of LPS in sepsis and endotoxemia. Interest in Gram-positive pathogens has heightened with the emergent of resistant pathogens such as methicillin-resistant *Staphylococcus aureus* (MRSA), leading to a push for a better understanding of Gram-positive sepsis. PG and lipoteichoic acids (LTA) are cell wall components that act as PAMPs and are associated with toxemia from Gram-positive pathogens. Injection of these molecules leads to many of the symptoms associated with Gram-negative endotoxemia, including fever. In equine models, different PAMPs can induce different inflammatory mediators, and synergy may exist when multiple PAMPs are administered together [42]. This has also been observed in human abdominal sepsis, where early cytokine profiles correlate to the Gram status of the infection [43]. While some signaling elements are common, pathways can vary substantially between Gram-negative and Gram-positive infections, as is discussed below.

### 3.1. Peptidoglycan and Lipotechoic Acid

Like Gram-negative LPS, Gram-positive pathogens possess PAMPs that can translocate into systemic circulation and activate the host immune system. PG makes up a large proportion of the dry mass of a Gram-positive bacterium, and forms only a small layer in Gram-negative bacteria underneath the outer membrane. PG can translocate into systemic circulation after bacterial lysis; a property originally detected using the silkworm larvae plasma test [44]. PG consists of alternating residues of N-acetylglucosamine (NAG) and N-acetylmuramic acid (NAM) which are linked in a β-(1,4) configuration, with strands crosslinked between tetrapeptide chains on NAM molecules. Most of the variation in PG from different species of bacteria lies in differences with the peptide chain. Crosslinking occurs directly in Gram-negative bacteria and via an amino acid bridge in Gram-positive bacteria. The third amino acid residue in these chains also varies. Gram-positive bacteria have L-lysine, while Gram-negative bacteria possess a meso-DAP residue. Modifications are common in terminal residues of the tetrapeptide chain, which results in antibiotic resistance. A substitution from D-ala to D-lac in the terminal dipeptide prevents vancomycin activity [45].

Also unique to the cell wall of Gram-positive bacteria, LTA traverses the Gram-positive cell wall to anchor in the bacterial membrane and has endotoxin-like activity. LTA from *S. aureus* consists of 1,3-polyglycerol phosphate attached to the Gram-positive membrane via a glycolipid anchor [46]. Structure can vary considerably across species, with changes in glycerol chain length, the glycolipid moiety and attached side groups being most common [47]. LTA serves a variety of functions in adhesion, ion scavenging and antibiotic resistance for the bacterium. It is known to activate the innate immune system, but has received considerably less attention as a toxin than either LPS or PG. It also plays a role in leukocyte migration, inhibiting the chemotaxis of neutrophils [48]. These properties have made LTA a priority for Gram-positive inflammation modeling.

### 3.2. PG and LTA Signaling

Gram-positive PAMPs signal through distinct pathways. Nucleotide-binding oligomerization domain-containing proteins (NODs) act as PRRs for innate immune recognition in the host cytosol and are the primary receptor through which peptidoglycan recognition occurs. Like TLRs, NODs recognize PAMPs via leucine-rich repeats (LRRs). The primary difference between TLRs and NODs is their location; TLRs are usually membrane-bound while NODs are cytosolic. For complete sensing of peptidoglycan by NODs, it must be broken down and internalized by phagocytes. NOD1 recognizes κ-D-glutamyl-meso-diaminopimelic acid, while NOD2 senses the muramyl dipeptide component of peptidoglycan. RIP2/RICK are recruited to the CARD domain of the NOD which subsequently activates NF-κB, a common endpoint of many PRR signaling pathways. Some controversy exists about peptidoglycan binding to TLR2. Variations in both peptidoglycan structure and purification methods may explain the discrepancies in study results [49]. Gram-positive and Gram-negative pathogens produce PG with both different structures and quantities. One study suggests TLR2 has more affinity for Gram-positive peptidoglycan, which has a higher lysine content, than Gram-negative PG, which may provide an evolutionary advantage by preventing immune overactivation [50].

Metabolic enzymes may also serve as PRRs for Gram-positive cell wall components. Internalization and degradation of pathogens by innate immune cells releases N-acetylglucosamine (NAG), a major component of peptidoglycan, into the cytosol of the immune cell. Hexokinase, a glycolytic enzyme present in the cytosol, is competitively inhibited by NAG and dissociates from the mitochondrial membrane to activate the NLRP3 inflammasome, which is a hallmark of Gram-positive infections. [51] This suggests a linkage between pathogen detection and metabolism. Wolff et al. showed that hexokinase mediates NLRP3 inflammasome-dependent secretion of IL-1β; a pathway distinct from classical inflammasome activators [51]. Additionally, reduced acetylation of NAG correlates with reduced activation as observed with de-acetylated peptidoglycan from *B. anthraxis* [51]. Glycolytic enzyme inhibition and inflammasome activation has also been observed with S. typhimurium [52].

Like LPS, LTA activates TLRs inducing a similar downstream pathway. Adaptor MyD88 is recruited to the TLR to induce proinflammatory cytokine production, as is described previously. TLR2 serves as a specific receptor for LTA. TRAM can serve as an adaptor protein for both LPS and LTA signaling in some cell lines, providing an alternative adaptor protein [53]. LTA toxicity can be enhanced by synergizing with hemoglobin to activate TLR2 and TLR4-dependent responses in macrophages [54]. Like with other toxins, variations in structure can impact the immune response. *Lactobacillus* are known to have variations in their LTA structures, which impacts their cytokine production via the MAPK pathway [55]. Interestingly, LTA from *Lactobacillus plantarum* can inhibit the immune effects of LPS, specifically reducing TNF-α production [56]. LTA can also induce nitric oxide production. Structural changes such as D-alanine substitutions and glycerol chain length modulate nitric oxide production in various *Bacillus* species [57]. In some initial studies some of the nitric oxide producing activity of LTA resulted from contaminating endotoxin in the commercial preparation [58]. This serves as a reminder to exercise caution when attributing new properties to a molecule under study. 

While significantly less is known about the role of Gram-positive toxins in sepsis, several studies have attempted to draw comparisons with Gram-negative sepsis. Based on differences in signaling, it is reasonable to expect some differences in immune response and microcirculatory changes, but the extent of this is largely unstudied. Yipp et al. compared leukocyte-endothelial interactions using intravital microscopy when administering LPS or LTA systemically [59]. Purified LTA resulted in a much weaker response than purified LPS, but the response to administration of live *S. aureus* was like that of LPS [59]. This suggests PG may be responsible for much of the immune response to Gram-positive pathogens. In addition to these known differences, some cross-reactivity exists between Gram-positive and Gram-negative PAMPs. Peptidoglycan recognition proteins (PGRPs) secreted by host cells bind to peptidoglycan on the surface of pathogens. These proteins also have variable affinity for other PAMPs, including LPS, LTA and mycolic acid [60]. Bacterial killing is mediated in several ways by these proteins, including the direct breakdown of PG bonds and the enhancement of immune recognition by phagocytes [49]. PGRP deficiency produced no phenotypic change in murine immune response, suggesting these proteins serve as an additional protective immune mechanism [61]. This cross-reactivity has also been observed in LPS-binding proteins (LBPs). LBPs have been shown to recognize the glycan backbone of the pneumococcal cell wall, suggesting an additional role in Gram-positive infections [62]. These redundant mechanisms provide an extra layer of protection for the host in case of a problem with primary response mechanisms. Knowledge of both differences in response and possible cross reactivity is essential for making direct comparisons between Gram-positive and Gram-negative toxins. 

Like endotoxin release by Gram-negative pathogens, the bioavailability of PG and LTA from *S. aureus* can be increased by β-lactam antibiotic administration, furthering inflammatory pathology by enhancing the release of pro-inflammatory cytokines [63]. Administration of protein synthesis inhibitor antibiotics had no impact on peptidoglycan release, but LTA release was reduced [63]. This data suggests careful consideration of antibiotics is necessary when treating infection regardless of the Gram status of the organism as toxin release is dependent on the mechanism of killing by the antibiotic. This provides further evidence that there may be an important role for non-antibiotic therapies in combatting these infections, which can be preliminarily assessed using endotoxemia models.

## 4. Selected Bacterial PAMPs

Bacterial cell lysis often releases other molecules that can act as PAMPs to trigger immune cell activation. Examples discussed include flagellin, porins, lipoproteins and heptose-1,7-bisphosphate. While these molecules are not strictly toxins, their ability to activate innate immune responses through various pathways merits some consideration for inflammation modeling. 

Bacterial flagellin is a conserved structure that can act as a bacterial PAMP. Flagellin is the primary protein component of the flagella of motile bacteria. Like previously discussed Gram-negative and Gram-positive pathogens, flagellin recognition is mediated by TLRs associated with bacterial membranes. TLR5 recognizes flagellin from both Gram-negative and Gram-positive pathogens, and signals through MyD88 [64]. Flagellin may also impact the microcirculation by enhancing leukocyte migration mediated by TLR5s expressed on microvascular endothelial cells [65]. *H. pylori* is a notable exception, as the altered amino acid sequence of its flagellin prevents immune recognition by TLR5, contributing to persistent infections that are often associated with *H. pylori* infection [66]. Various NAIPs (NOD-like receptor apoptosis inhibitory protein), which are NOD-LRRs in the inflammasome, detect cytoplasmic flagellin and components of bacterial type 3 secretion systems, responsible for injection of virulence proteins, to mediate caspase-dependent immune responses. Multiple isoforms of human NAIP allow detection of the inner rod, needle and flagellin for various bacterial species, mediating an immune response by the NLRC4 inflammasome [67]. 

Various bacterial porins have been identified as able to activate TLR signaling. They may act as a PAMP both alone and cooperatively with LPS. TLR2 recognizes *Shigella flexneri* porins and triggers upstream NF-κB activation as with LPS administration via convergent pathways, but differential cytokine production occurs [68]. Neisserial porins also signal in a TLR2 dependent fashion [69]. *Vibrio cholera* porin OmpU is pro-inflammatory but has been demonstrated to downregulate the pro-inflammatory TLR response to LPS leading to tolerance [70]. Aquaporins, specialized for water transport, have an established regulatory role during sepsis. Specific aquaporins may be upregulated or downregulated in response to LPS administration suggesting they may play a role in sepsis pathogenesis [71]. LPS, shiga toxin and cholera toxin have all been shown to alter aquaporin expression [72,73]. Aquaporins can enhance immune cell migration via water influx to facilitate actin remodeling at the leading edge of the cell; like AQP9 acting in neutrophils [74,75]. AQP1 expression is known to be upregulated in human sepsis, likely due to LPS-triggered activation [71]. Current literature suggests porins could be considered for sepsis modeling in combination with LPS, or alone. 

Bacterial lipoproteins act as a bridge between the PG and the outer membrane in Gram-negative bacteria. In Gram-positive bacteria, lipoproteins are in the periplasmic space. These relatively conserved protein structures can act as bacterial PAMPs, signaling through TLRs. Lipoproteins are recognized primarily by TLR2, but TLRs 1, 6 and 10 are also involved in the formation of heterodimers with TLR2 for the discrimination of specific lipoproteins. TLR1 and TLR6 are known to interact with TLR2 to distinguish between triacyl and diacyl lipoproteins of *Mycoplasma* respectively [76]. LPS and lipoproteins can act synergistically, producing cytokines via separate pathways, which suggests lipoprotein may play an important role in sepsis [77]. Additionally, pre-treatment of mice with lipoprotein induced cross tolerance to a subsequent lipoprotein challenge, and to challenges with both LPS and whole bacteria in endotoxemia and cecal ligation and puncture models respectively [78]. This tolerance is mediated by both TLR2 and TLR6 in murine macrophages [79]. Partial cross tolerance to Gram-positive sepsis can be induced by LPS challenge, suggesting some common elements in activation pathways of other Gram-positive PAMPs [80]. No evidence exists that LPS challenge provides tolerance to lipoproteins specifically. 

Other cytosolic PAMPs are produced by some bacteria. Some Gram-negative bacteria express heptose-1,7-bisphosphate (HBP), which is a core intermediate for LPS synthesis and a component of Gram-negative cell walls. HBP is known to be transferred to the host cell via a Type IV secretion system in *H. pylori*, activated by cell to cell contract, where it can then activate NF-κB from the cytosol. HBP from *Shigella* and *Neisseria* have also been shown to act as an innate immune agonist via interactions with receptor alpha-kinase 1 (ALPK1) [81,82]. ALPK1 then engages the host Traf-interacting protein with the fork head-associated domain (TIFA) signaling pathway leading to NF-κB-dependent immunity against Gram-negative bacteria [83].

## 5. Conclusions

Endotoxemia experiments serve as front line experiments in the field of sepsis research as they allow the examination of specific bacterial PAMPs, and the elucidation of specific pathways of inflammation. The role of LPS in sepsis has been well established due to its extensive usage in endotoxemia studies. Gram-positive PAMPs have received significantly less investigation historically but have recently emerged as a topic of interest in sepsis. An understanding of signaling and pathways to inflammation in response to the various PAMPs produced by both Gram-negative and Gram-positive bacteria is essential to developing effective treatments. This review has addressed various toxins and bacterial PAMPs, including LPS, peptidoglycan, LTA, and several other molecules which act as immune system activators. Agents for inflammation modeling should be selected based on the pathway or mechanism of interest, as discussed above. Combinations of PAMPs may provide a more physiological response than individual PAMPs alone, providing insight into possible synergy and cross tolerance between PAMPs. Pathogen response can vary significantly, so the specific pathogen, PAMP, and the concentration given must be considered when selecting appropriate agents for a novel study. Possible variations between the model animal and human immune responses must also be considered. Despite potential challenges, the versatility and adaptability of murine endotoxemia models makes them an important aspect of sepsis research.

## Abbreviations

LPS	Lipopolysaccharide
PG	Peptidoglycan
LTA	Lipotechoic acid
PRR	Pattern recognition receptor
PAMP	Pathogen-associated molecular pattern
KDO	C-8-keto-deoxy-octonic acid
S-LPS	Smooth lipopolysaccharide
R-LPS	Rough lipopolysaccharide
TLR	Toll-like receptor
MD-2	Myeloid-differentiation factor 2
mCD-14	Membrane-associated CD-14
MAPK	Mitogen-activated protein kinase
NAG	N-acetylglucosamine
NAM	N-acetylmuramic acid
MRSA	Methicillin-resistant *Staphylococcus aureus*
NOD	Nucleotide-binding oligomerization domain-containing protein
LBP	Lipopolysaccharide-binding proteins
NAIP	NOD-like receptor apoptosis inhibitory protein
HBP	Heptose-1,7-bisphosphate
ALPK1	Alpha-kinase 1
